# More about Rigg's Disease

**Published:** 1880-08

**Authors:** George A. Mills

**Affiliations:** Brooklyn, N. Y.


### ARTICLE II.

### More About Riggs Disease.

BY GEORGE A. MILLS, BROOKLYN, N. Y.

(Read before the Con. Valley Association, June 17, 1880.)

*Mr. President and Gentlemen*:—I deem it not unprofitable to present to you particularly, what I have prepared regarding a subject that I may say truly, that you have been the instrument of bringing into somewhat an extended notice to our profession, while the part you have taken in this matter has aroused not a little antagonism, arising from a variety of motives which are common among men, and have at times disturbed some, who thought perhaps their particular modes and practices impregnable; and all we can ask of them "who have always done it" is to join with the progressive movement that has been put into action and rejoice in the good that may accrue from the results of this beneficial practice, which brings so much relief to impaired physical conditions prevalent among those we are called upon to serve. You are aware of the part I have taken in this controversy. I have abundant testimony that I have not stated anything that was not true and is not now fully proved beyond controversy to the understanding of any who seek the *unbiased* truth. When I published my five articles in Cosmos, titled: "What I know about Riggs Disease," I did better than I thought. For the first time in the meeting of the American Dental Association, the following August at Chicago, the subject gained the attention of our profession by an extended discussion, which you will find published in their proceedings, numbering some twenty-seven pages, and on my reading them I was confirmed in thought, that it only needed fair and dispassionate attention to impress all true men, that it required and demanded an earnest investigation in the interest of our clientage. Some eighteen months later at a meeting of the Brooklyn Dental Society the subject of popular education was discussed and the necessity was indicated, that it needed to be done, to make it useful, in a more efficient manner. A remark was dropped that what the public needed was truth on points that were essential to their interest. The impression came to me then, that I had in those articles something which was to their interest to know, and coupled with the thought, it occurred to me that a larger dissemination of the same would not do any harm among the profession. The result of this was the publication of my little brochure under the title " A Series of articles on the so-called Riggs Disease," "loosened teeth" or Pyonhora Alveolaris; [pus discharging sockets of teeth] which some of you have seen. Taking the view of this movement that I did, I was not at all troubled about being disturbed. I commenced their distribution not only among some of my patients as opportunity offered, but I mailed a copy to several members of the profession with whom I had personal acquaintance and soon much to my gratification [but quite unexpected] I received commendatory letters regarding them, and also a pleasant notice was given in the Dental Register by Professor Taft, saying no doubt I would gladly on the receipt of postage send one to any desiring them. Suffice it to say a continued distribution has been made up to this time numbering some seven hundred among the profession throughout this and foreign countries, and it has been the means of bringing me into correspondence with many, affording both pleasure and profit. I was also much gratified to find Dr. Riggs was pleased to endorse what I had written and that he has since given a free distribution of the articles among his patients and this has also been done by dentists. The amount of discussion growing out of the agitation of this subject has not been small among our associations and has also taken on no little interest among those of other countries; we have testimony of these facts on the pages of our several journals. This subject is still worthy of the attention of a greater number of our profession, than those having yet given it, for it is far from becoming an actual daily practice with many and those who talk about it even, giving an impression which does not accord with the condition of the mouths of some of their patients. I could detail a good many specimen cases; there are those who talk much to their patients, but utterly fail in practice; I will give you an instance. My attention was called to a case that had been under treatment some six months. I found all the teeth discharging pus. A superior central incisor was quite a good deal elongated and by occlusion of the teeth, it was made to play in and out, keeping up a continuous state of unrest which of course would be but a matter of time how long it could remain in its socket, certainly without removing the mechanical interference by grinding so that it could be brought into its proper position and fastened by ligatures or otherwise. It has been my observation that such a failure of understanding of these cases is not an isolated one, and my calling attention to the fact may of itself be suggestive to the ingenuity of any that should wish to make a greater effort than they had hopes of doing with success. There are many cases of this kind presenting themselves to the practitioner with not only one tooth disturbed by improper occlusion, but often several. The ligatures, elastics, silk, wire, disks, and the new cast Gold Alloy are valuable auxiliaries for the control of such conditions. I shall refer, further on, to this matter and introduce to your notice an advance line of practice giving additional success to the treatment of loosened teeth in a greater degree than has ever before been secured. This diseased condition of the surroundings of the teeth has called considerable attention to the causes, and opinions have been given by different ones and still they are thought to be somewhat obscure, and I trust what I may say on this point, may not wholly fail to contribute something helpful.

Physiology is the building of tissues. Pathology the unbuilding. That we may be able to comprehend something of the service committed to our hands it will be of value to take into consideration the etiology of the subject we purpose to consider; first we are asked what is the cause of this destructive disease? (Riggs Disease.) I answer, debility. Some say the collapsing of the margin of the pericementum, causing it to drop back from its surrounding of the hard tissue, the neck of the tooth; others, that the deposition of lime carried by the saliva forming a crustlike collection and this acting as an irritant, setting up inflammatory action. I have given my answer that debility is the general cause and I may add very properly that it is "in esse" (in being) and local in manifestation. We have the fact before us, that, if the margin of the pericementum has commenced to disintegrate inflammatory action has already commenced its work; we also have the same evidence of debility by the deposits of lime; from one class of ducts we have an acid secretion; another, alkaline; this gives the saliva to the oval cavity in equilibrium; anything that may come in as a disturbing element to the nutritive action of the general system will cause the unbalancing of this satisfied solution and produce a precipitative action, indicating an abnormal condition. What are the working powers of tissue building into organizations such as ours? I answer, the *sensory*, and *motory* nerves; every influence favorable or adverse to these will cause a decided deflection by bringing about a state of exaltation or depression; this latter is exhibited in a marked degree in different parts of the human system, where the morphology (type or form) is seen to be deficient; as an illustration of this we see in the teeth with their impoverishment or want of calcification, a decided deficiency in the cusps and other parts, also expressed by a change in the saliva caused by a fright or anger, also, by exultation in joy, the sight or smell of food. Now it is readily seen that the nervous system is exposed in a multitude of ways from without and within, and with our present surroundings we cannot hope to escape entirely from their effects. This disturbance at the margin of the pericementum is undoubtedly originated by lack of circulation through the capillaries. When the diastolic and systolic action is enfeebled by continued disturbance, this forcing power falls short of its intended work. In other words, when the tonicity of the nerve force is deficient, we see debility; this produces a decrease of rension and is readily seen in its effects on the action of inspiration and aspiration of the lungs, by shortening the lengths of the breathings, by short breath, less oxygen is carried into the circulation, and stimulation of the blood current is lessened and the parts that require given amount of power to force the circulation to them, suffer by this enfeebled condition and they are from a want of sufficient nourishment reduced back to their embryonal condition. At this stage we find the work of reparation brought into requisition. The different degrees of this destructive disease are quite familiar to many. At this point let me suggest to every earnest dentist that he become familiar by *repeated readings* with the very exhaustive articles prepared by Dr. Bodecker, an honest and earnest investigator in the laboratory of Dr. Carl Heitzman of New York City. These papers are too valuable to be passed by lightly; they contain a condensed amount of instruction that should arouse the gratitude of *every dental student*, and I think the best way it could be manifested to the author, would be by showing a familiarity with his teachings. One particular point I will call attention to in these papers, viz: the finding of abscesses in connection with the inflammation of the pericementum and not associated wiih a dead pulp. This is comparatively a new presentation of this matter; I have in a number of cases come in contact with them and have been spoken to about them by other practitioners, and you have doubtless noticed reference to the fact by others in our journals of late. These conditions are complications of the disease which we are considering and need be borne in mind together with the treatment looking to a successful issue. Regarding the methods that have been presented in the treatment of this disease, I have nothing more to say about them than I have said, but I purpose at this point, to direct your attention to an additional line of treatment which brings to the aid of the practitioner a degree of helpfulness that has not before been attained and secures to the patient a success and amount of personal comfort that is truly gratifying; the cases are frequent when the teeth are deprived of part or parts of the socket supports and also by the loss of teeth adjoining them, and thus depriving these of their lateral supports, changing the occlusion by a general pushing out of line. You are familiar with such a state of things. If you will consider (for the moment) the value of an unbroken arch (on general principles) you will discover the importance of a lost tooth. How many are the cases that exhibit this disturbed and irregular condition? Is the effects of misapplied mechanical force by the changed occlusion, muscular tension and absorption of the soft and hard tissues, all these are disabilities and additional enhancements of the difficulties connected with the disease under consideration. While much can be done for the general improvement of the health and personal comfort of the patient by the before prescribed methods, we are now able to bring the teeth out of line and arch into their proper position, by ligatures, elastics and wires to remove mechanical interference from elongation and then by supplying the deficient lost tooth, combined with the cast new base (Gold Alloy) all together making such a combination of framework, giving a support to these loosened teeth, enabling the patient to put their previous *useless masticators* into comfortable use, from the instant, and giving an additional assurance that nature will be better able to supply the lost tissue surrounding the teeth by the state of rest secured with these auxiliaries. I am now speaking of the results gained by actual practice and observation both in Dr. Atkinson's and my own office; to Dr. Atkinson I am indebted for this advanced step. Now I am conscious that the question has already arisen, "What are you able to secure in the application of the new base more than by any other we have been using?" First, a greater degree of compatibility to the tissues (doubtless) in a comparative sense than from any other known material. Second more adaptation to the form of the plate resting on the soft tissues and the surroundings of those parts of the teeth requiring the support of the plates laterally and otherwise. A patient securing the intelligent treatment of the destructive disease, (Riggs Disease,) inflammation of the pericementum, or what other name you may choose to apply to it, all supply of deficiencies of the hard tissues by fillings, the appliance combining the lost teeth and its mechanical support, gives a complete, useful and healthful oval cavity into their possession under such a sense of appreciation of the value it possesses, gained by the earnest spirit of labor manifested by him who has performed it, as to permit of no future departure to the lower level from which they have been brought; and more, it sends out into their circle of association an educator, with a degree of potency, that will excelerate an upward tendency and do more to prove the value of dentistry in a much higher degree than has ever before been attained.—*Independent Practitioner.*

